# Laser-Induced Silver Nanowires/Polymer Composites for Flexible Electronics and Electromagnetic Compatibility Application

**DOI:** 10.3390/polym16223174

**Published:** 2024-11-14

**Authors:** Il’ya Bril’, Anton Voronin, Yuri Fadeev, Alexander Pavlikov, Ilya Govorun, Ivan Podshivalov, Bogdan Parshin, Mstislav Makeev, Pavel Mikhalev, Kseniya Afanasova, Mikhail Simunin, Stanislav Khartov

**Affiliations:** 1 Federal Research Center, Krasnoyarsk Scientific Center, Siberian Branch, Russian Academy of Sciences (FRC KSC SB RAS), 660036 Krasnoyarsk, Russia; a.voronin1988@mail.ru (A.V.); daf.hf@list.ru (Y.F.); shabanova.ksenia@mail.ru (K.A.); michanel@mail.ru (M.S.); stas_f1@list.ru (S.K.); 2 Department of Radio-Electronic Systems and Devices, Bauman Moscow State Technical University, 105005 Moscow, Russia; parshbgal@bmstu.ru (B.P.); m.makeev@bmstu.ru (M.M.); pamikhalev@bmstu.ru (P.M.); 3 Siberian Federal University, 660041 Krasnoyarsk, Russia; hahanka@yandex.ru; 4 Kirensky Institute of Physics, Federal Research Center KSC SB RAS, 660036 Krasnoyarsk, Russia; govorun-ilya@mail.ru (I.G.); podshivalov.ivan@gmail.com (I.P.); 5 Reshetnev Siberian State University of Science and Technology, 660037 Krasnoyarsk, Russia; 6 Institute of Space Technologies, FRC KSC SB RAS, 660037 Krasnoyarsk, Russia

**Keywords:** flexible electronics, silver nanowires, sheet resistance, shielding efficiency

## Abstract

Nowadays, the Internet of Things (IOT), electronics, and neural interfaces are becoming an integral part of our life. These technologies place unprecedentedly high demands on materials in terms of their mechanical and electrical properties. There are several strategies for forming conductive layers in such composites, e.g., volume blending to achieve a percolation threshold, inkjet printing, lithography, and laser processing. The latter is a low-cost, environmentally friendly, scalable way to produce composites. In our work, we synthesized AgNW and characterized them using Ultraviolet-visible spectroscopy (UV-vis), Transmission electron microscopy (TEM), and Selective area electron diffraction (SAED). We found that our AgNW absorbed in the UV-vis range of 345 to 410 nm. This is due to the plasmon resonance phenomenon of AgNW. Then, we applied the dispersion of AgNW on the surface of the polymer substrate, dried them and we got the films of AgNW.. We irradiated these films with a 432 nm laser. As a result of the treatment, we observed two processes. The first one was the sintering and partial melting of nanowires under the influence of laser radiation, as a consequence of which, the sheet resistance dropped more than twice. The second was the melting of the polymer at the interface and the subsequent integration of AgNW into the substrate. This allowed us to improve the adhesion from 0–1 B to 5 B, and to obtain a composite capable of bending, with radius of 0.5 mm. We also evaluated the shielding efficiency of the obtained composites. The shielding efficiency for 500–600 nm thick porous film samples were 40 dB, and for 3.1–4.1 µm porous films the shielding efficiency was about 85–90 dB in a frequency range of 0.01–40 GHz. The data obtained by us are the basis for producing flexible electronic components based on AgNW/PET composite for various applications using laser processing methods.

## 1. Introduction

Flexible electronics have become a common part of our life. Radio Frequency IDentification (RFID) tags, neural interfaces, and wearable sensors have all been included in our lives thanks to the impressive rate of development in this industry. In addition to targeted applications as electronic components, flexible electronic materials can be used as electromagnetic compatibility components. With the development of the flexible electronics industry, the demand for flexible shielding materials has also increased. Flexible electronics, just like traditional ones, consists of three main types of components: 1—substrate; 2—conductive paths; 3—the basic components of which the electronic circuit consists [1].

Currently, polymer materials are used as substrates. There are several reasons for this: polymers are inexpensive to produce and there is a possibility to recycle them, and creating conductive paths is one of the most important tasks in the manufacture of flexible electronics. We can formulate the requirements for this element: 1—it must have the lowest possible resistance; 2—it must be durable and retain its properties under various mechanical influences; 3—it must retain its electrical properties under various bending conditions.

The most advanced technologies make conductive paths and patterns. There are various types of lithography and printing with electrically conductive inks [2]. These inks are dispersions of conductive agents in liquid. Types of inks can be categorized by the type of conductive agents. These can be: 1—carbon materials, such as carbon nanotubes [3,4], carbon black [5], and graphene [6]; 2—modern nanomaterials such as MXenes [7] and other materials [8]; 3—metal materials, such as metal nanoparticles [9] and metal nanowires [10]. Nanoscale conductive agents are preferred, as they allow an increased resolution of printing [11]. The point is that inkjet printing allows the creation of conductive paths with good homogeneity but does not contribute in any way to the adhesion of conductive paths to the substrate. As a result, such paths may not withstand mechanical stress without additional protection. Additionally, such methods are neither inexpensive nor environmentally friendly and they are technically challenging because they require the use of preformed masks [12]. In recent years, laser-based approaches to creating flexible electronics have become increasingly popular as cost-effective, environmentally friendly, and low-cost alternatives to traditional methods [13,14,15]. Some examples of the amazing results of this approach can be found in the results of Liu and Lee in the formation of silver templates on polyimide substrate [16], the sintering of silver nanoparticles on polyethylene terephthalate (PET) substrate by Kim et al. [17], and the laser-formed composite of aluminum nanoparticles and PET shown in the work of Rodriguez et al. [12]. These and other works related to laser processing of nanomaterials/polymers inspired us to expand our approach to creating electrically conductive nanocomposites using the silver nanowires (AgNW). We expect silver nanowires to act as a photothermal converter due to the effect of localized surface plasmon resonance in the UV-blue wavelength [18], which will make it possible to create a conductive composite of AgNW and any thermoplastic polymer using laser processing. Such composites must have phenomenal wear resistance, and their flexibility will be determined by the mechanical properties of the substrate. To test this hypothesis, we synthesized low aspect ratio nanowires, deposited films on thermoplastic polymers, laser treated them, and assessed their optical, electrical, and mechanical properties. The results obtained will be a significant addition to the rapidly developing topic of laser-induced conductive composites.

## 2. Materials and Methods

### 2.1. Synthesis of AgNW

AgNW were synthesized by the following method. First, 1.3 g polyvinylpyrrolidone (PVP) (100 kDa) (ITW Reagents Panreac Castellar del Vallès, Barcelona, Spain) was dissolved in 40 mL of ethylene glycol (EG) (EKOS, Moscow, Russia) into three-necked flask. The flask was slowly heated to 160 °C with vigorous magnetic stirring. When the temperature had stabilized at 160 °C for 1 h, an ethylene glycol solution of FeCl_3_ was added. Then, 10 mL of 1.47 M ethylene glycol solution of AgNO_3_ was added to the flask. Then the flask was sealed until the solution became glistening, indicating the formation of AgNW [19]. After the synthesis, we replaced the dispersant with AgNW. For this, the resulting dispersion in ethylene glycol was centrifuged at 10,000 rpm for 15 min using a laboratory centrifuge OPN 16 (Labtex, Moscow, Russia). After centrifugation, the supernatant was drained, and the same amount of isopropyl alcohol was added. This operation was repeated 5 times until the complete removal of ethylene glycol, NO^3−^, Cl^−^, Fe^3+^ ions, and PVP residues.

### 2.2. Samples Preparation

We used polyethylene terephthalate (PET) (Hi-Fi Industrial Film Ltd., Stevenage, Hertfordshire, UK), polyethylene naphthalate (PEN) (Toray industries, Tokyo, Japan), and polyvinylidene fluoride (PVDF) (3 M, Saint-Paul, MN, USA) as substrates for the application of films by drip casting. We applied different volumes of base dispersion (~20 gm/mL concentration): 12.5, 25, 37.5, 50, 62.5, and 75 µL/cm^2^.

### 2.3. Microscopy

Optical microscopy photographs were taken on an Altami 104 microscope (Altami, Saint Petersburg, Russia). The morphology and geometric characteristics of individual AgNW were studied by transmission electron microscopy (TEM) on a HT 7700 (Hitachi, Tokyo, Japan) at an accelerating voltage of 40–300 kV. The morphology of AgNW films was studied by scanning electron microscopy (SEM) on a SU3500 microscope (Hitachi, Japan) at an accelerating voltage of 20 kV. EDX images and spectra were obtained on a SU3500 (Hitachi, Japan) microscope equipped with an energy-dispersive X-ray spectrometer XFlash 430 (Brucker, MA, USA).

### 2.4. XRD

X-ray diffractograms of the samples were taken using a X’Pert Pro MPD diffractometer (PANalytical, Almelo, The Netherlands) with a high-speed PIXcel detector in the angular range 30–90° 2Θ, with a step of 0.013°. The Ag lattice parameters were determined and refined using the full Rietveld approach by the difference derivative minimization (DDM) method.

### 2.5. Optical Properties

The optical density of AgNW was measured in the range of 280–800 nm using a UV-3600 spectrophotometer (Shimadzu, Kyoto, Japan). For this purpose, the base dispersion of AgNW with a concentration of ~20 mg/mL was diluted 100 times. Then, 1 mL of the obtained diluted dispersion was poured into a quartz cuvette. The spectrum was taken with preliminary acquisition of the baseline of pure dispersant (ethanol).

### 2.6. Electrical Properties

The sheet resistance of AgNW films was measured by the four-probe method using a JG ST2258 four-point probe station (Suzhou Jingge Electronics Co., Suzhou, China) and a JG ST2558-F01 four-probe head (Suzhou Jingge Electronics Co., Suzhou, China).

### 2.7. Shielding Efficiency

The S_21_ and S_11_ coefficients of the materials were measured by the waveguide method. The investigated sample was placed in the break of a waveguide transmission line.

This investigation used a wide range band (0.01–7 GHz) and a special air coaxial cell with a diameter of 16.00/6.95 mm (type II, 50 Ω, GOST RV 51914-2002). The measurements were carried out in the range of 10 MHz to 7 GHz; this frequency range includes the L (1–2 GHz), S (2–4 GHz) and C (4–8 GHz) bands. The ability to measure at low frequencies with a relatively simple and convenient measurement technique provides good quality results. The measurements were carried out on a Keysight FieldFox N9916A vector network analyzer (Keysight Technologies, Santa-Rosa, CA, USA).

K band (18–26.5 GHz) and a Ka band (26.5–40 GHz) were used. The waveguide-coaxial transitions had a rectangular cross section with dimensions of 4.3 mm × 10.65 mm for K band and 3.55 mm × 7.1 mm for Ka band. Measurements were performed on an R&S ZVA 40 vector circuit analyzer (GmbH & Co. KG, Großheringen, Germany).

### 2.8. Mechanical Properties

The adhesion strength of AgNW/PET composites was evaluated using a tape test according to ASTM D 3359, in geometry B (notch lattice period was 2 mm). The effect of the reusable tape test on resistance AgNW/PET composites was also studied. In all studies, 3 M tape was used, and the test sample was firmly fixed on the table. The mechanical properties of AgNW/PET composites were studied in comparative experiments on a laboratory bench in single load and cyclic modes [20]. For the single bend mode, we took several bending radii: 10 mm, 5 mm, 2 mm, 1 mm, and 0.5 mm. The bending templates were fabricated using 3D printing. Cyclic bending allowed us to investigate fatigue accumulation in AgNW/PET composites. In our experiments, the number of bending cycles with a radius of 0.5 mm was 1000.

## 3. Results

### 3.1. AgNW Characterization

**Figure 1:**
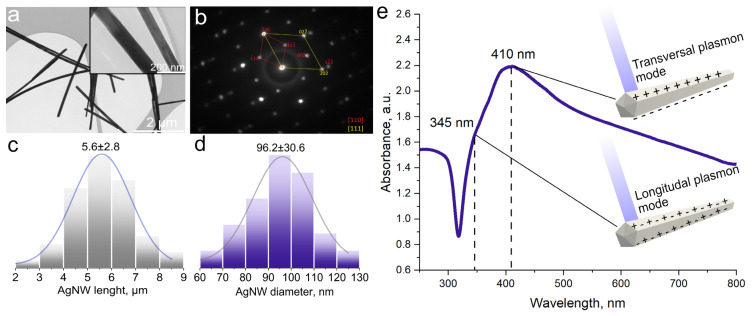
AgNW characterization. (**a**)—TEM Image, (**b**)—SAED, (**c**)—AgNW diameter, (**d**)—AgNW length, (**e**)—UV-vis absorption spectra.

We used TEM, selective area electron diffraction (SAED) and UV-Vis spectrophotometry techniques to study the structure and morphology of AgNW. Figure 1a shows the TEM image of the resulting AgNW.

**Figure 2:**
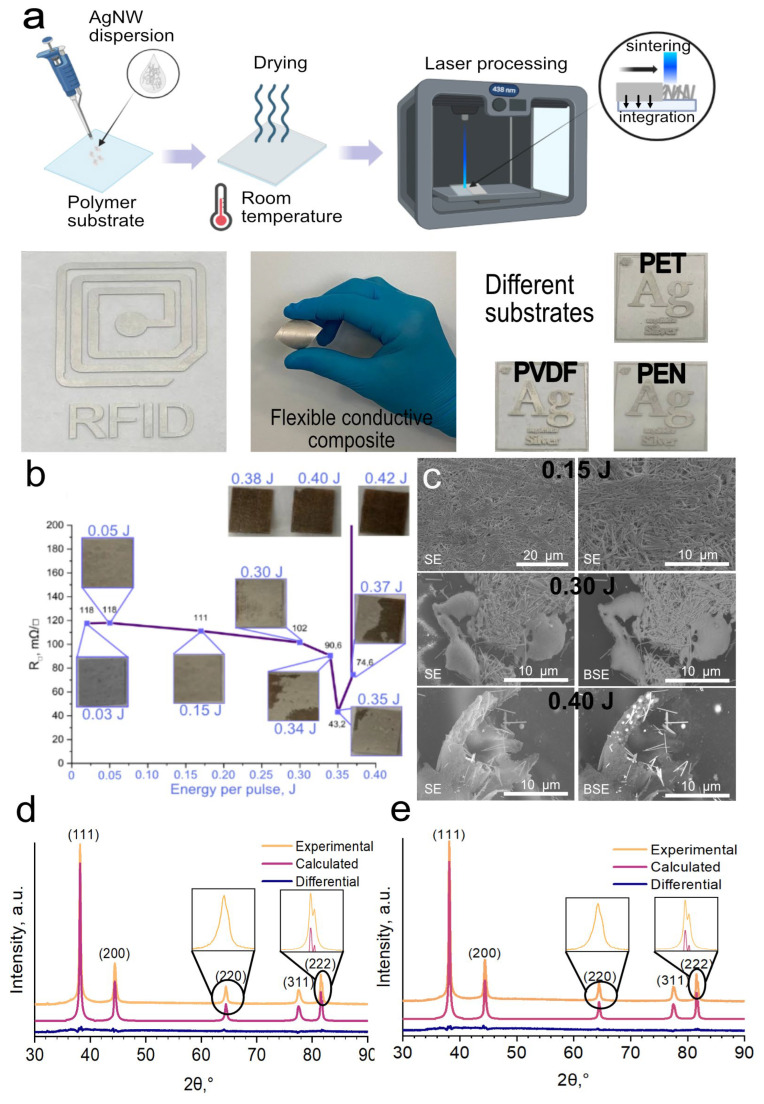
Samples preparation and characterization. (**a**)—Samples obtaining scheme, (**b**)—effect of power density on electrical parameters and morphology, (**c**)—SEM images at different power densities, (**d**)—XRD of original film, (**e**)—XRD of laser-processed film.

Knowing the average AgNW length (5.6 ± 2.8 μm) and average AgNW (96 ± 30.6 nm) (Figure 1c,d) diameter, we can calculate the average aspect ratio–58.3. Using electron diffraction on the selected region, we see the presence of reflections 111 and 002 which show that the AgNW have more than one silver single crystal, i.e., on the twin structure. The fivefold twinning indicates that we have nanowires with a non-symmetrical pentagon in the cross section. (Figure 2b).

We see a peak and a satellite on the UV-vis absorption spectrum (Figure 1e). They are related to the phenomenon of surface plasmon propagation in nanowires [20,21]. Twinization is a typical growth behavior of nanowires, often found in the literature [22,23]. The complexity of the shape of the plasmon absorption peak increases with the decreasing symmetry of the pentagon in the cross section. We see a peak and a satellite on the UV-vis absorption spectrum. The peak maximum is at a wavelength of 410 nm. This is different from most results found in the literature [24,25]. According to Todd’s work, the absorption peak moves towards the blue–green wavelength region with increasing AgNW diameters, [26] which is what we see in our case. A surface plasmon can heat AgNW to 1000 °C, which is higher than the melting point of silver [27]. This, as well as the high degree of absorption in the blue wavelength region, inspired us to attempt laser-induced sintering of nanowires and their simultaneous integration into a thermoplastic polymer substrate with a blue microsecond laser.

### 3.2. AgNW/Polymer Flexible Composites Obtaining and Characterization

We used a dispersion of AgNW in ethanol, with a concentration of ~20 μL/cm^2^, to deposit AgNW on a polymer substrate using a drop-casting method. We chose polyethylene terephthalate (PET), polyvinylidene fluoride (PVDF), and polyethylene naphthalate (PEN) as substrates. After AgNW deposition, our films were dried at room temperature for 24 h. To make conductive free-form patterns on substrates we used a diode laser with 438 nm wavelength. The process scheme can be seen in Figure 2a. This process is possible for any thermoplastics. We have fabricated conductive patterns on commercial PEN and PVDF (Figure 2a). We chose PET as a substrate for further research as it is one of the most used polymers.

The quality of the composite can be varied by modifying two main parameters: the thickness of the AgNW coating and the amount of energy transferred per laser pulse.

It is possible to consider the influence of these two mechanisms on the target parameters of the composite separately. The selection of suitable laser processing parameters for the formation of laser-induced composites is a critical step. By provoking surface plasmon excitation, silver nanowires can reach high temperatures, which can provoke various phenomena occurring with the polymer substrate. The first is melting the nanowires and sintering the contacts. This affects the mechanical and electrical properties of the composite and is described in detail in the sections on electrical and mechanical properties. The second process is the melting of the polymer at the polymer/AgNW interface and the integration of the nanowires into the polymer. The third process is the pyrolysis of the polymer substrate, occurring due to excess energy received from laser radiation.

In Figure 2b, we can see the effect of laser irradiation energy on the morphology and electrical properties of the AgNW/PET composite. With increasing energy, we can see a trend of decreasing resistance. This is due to the sintering of nanowires and, as a consequence, the increase of the contact spot between nanowires (Figure 2c). For example, with increasing energy from 0.03 J/pulse to 0.35 J/pulse, we see a drop in resistance from 118 to 43 mΩ/sq However, further increase of energy leads to pyrolysis of the substrate and intensive melting of nanowires. For example, at an energy of 0.40 J/pulse we obtain a fully pyrolyzed region with pronounced carbon structures, inside which are integrated AgNW globules formed after melting (Figure 2c). Such phenomenon of Ag nanoscale objects is described by Rodriguez et al. [12].

An important parameter for flexible electronics manufacturing is reproducibility. Figure 2b shows that after the energy transition to 0.30 J/pulse, the reproducibility even on small areas becomes not satisfactory. This is due to the density heterogeneity of the films. Highly concentrated areas absorb laser radiation and heat up more than low concentrated areas, causing local pyrolysis of PET.

Taking this into account, it is reasonable to determine the production of subsequent samples at a power insufficient to cause the undesirable effects of the imperfections of the drop-casting method but that allows the minimum resistance to be obtained, which is 0.30 J/pulse.

For a more detailed investigation of the processes occurring during laser sintering and composite formation, we compared the XRD spectra of the original and laser-treated films. In Figure 2d,e we see peaks attributed to metallic Ag. Five distinct diffraction peaks at 2θ = 38.13, 44.4, 64.4, 77.47, and 81.5° were indexed to the (111), (200), (220), (311), and (222) reflections of metallic Ag. Ag in our case has a tetragonal distorted lattice and parameters: a = b=4.088(1), c = 4.095(2) Å. This indicates the preservation of the crystalline structure of AgNW after exposure to 0.30 J/pulse (0.19 J/cm^2^), which is in agreement with the literature on laser welding and sintering of AgNW [28,29].

### 3.3. Laser Sintering AgNW/ PET Composite Morphology and Electrical Properties

To study the property of our obtained AgNW films, we fabricated 12 samples with six different thicknesses on glass substrate and PET, respectively.

The thickness is an important parameter. Changing the thickness allows the indirect changing of the amount of energy delivered per unit area per impulse [30,31]. Recent literature has reported the influence of the thickness of metallic nanoparticle and nanowire films. Reducing the thickness of the layer has a positive effect in most cases, as it allows more energy to be delivered per unit volume, resulting in a more uniform sintered film, reduced porosity, and improved electrical and mechanical parameters. [31,32].

The reduction of porosity and free volume in the AgNW layer can influence the thickness of the AgNW layer obtained after laser treatment. In order to study this, we deposited six different thicknesses of AgNW films on a glass substrate, after which we split the sample into two parts and looked at the cross-section in SEM, as well as taking the EDX spectra. Glass was chosen as the substrate because it is much easier to split it into two parts than any thermoplastic polymer substrate.

**Figure 3:**
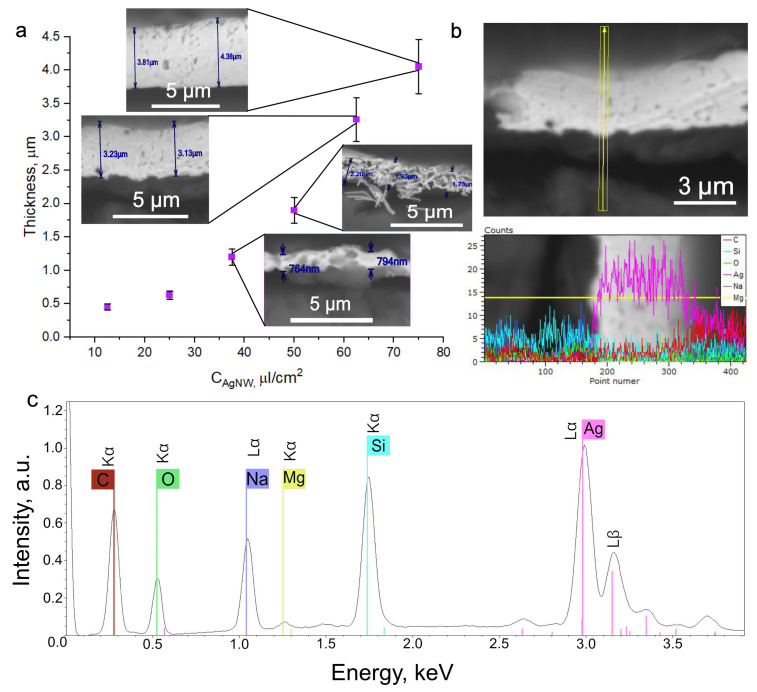
Thickness effect study. (**a**)—Dependence of thickness from concentration of AgNW basic dispersion, (**b**)—SEM image of x-section of 62.5 μL/cm2 BSE (top image), (**c**)—EDX spectra.

Figure 3a shows the concentration dependence of the layer thickness of irradiated nanowires. Taking into account that small concentrations of the basic dispersion give low homogeneity of the coating, we took several points and calculated the average thickness. As we can see, the dependence can be described by a straight-line equation, but low concentrations (12.5 and 25 μL/cm^2^) do not fit the trend due to the high inhomogeneity of the layer.

In Figure 3b we see the EDX of the AgNW film, with a concentration of 62.5 μL/cm^2^. At the top we see the scan area of the sample in the BSE picture, at the bottom the intensity of the element peaks of each mapping point. We see the expected dominance of silver intensity in the region related to the irradiated AgNW film, in the other regions we see the presence of spectra of silicon, oxygen, and sodium, which are components of the glass substrate.

In Figure 3c we can see the EDX spectra. They clearly show the X-ray series of the silver spectrum corresponding to the laser-irradiated AgNW film. In addition to them, we can also see a series of spectra of the glass substrate components (Si, Na, O, etc.).

The most important characteristic for materials used in flexible electronics applications is the electrical resistance of the material. In our case, the conductive layer is a thin integrated AgNW layer.

Usually, such samples are characterized by the sheet resistance, which is the resistance of a rectangular surface with a certain thickness [33,34].

**Figure 4:**
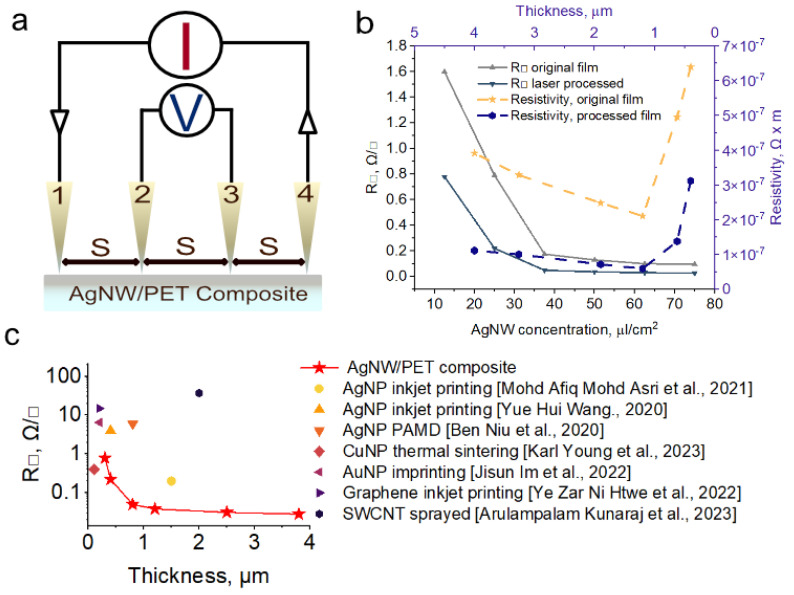
Electrical properties. (**a**)—Four-point probe measurements illustration, (**b**)—sheet resistance/thickness dependence and resistivity of AgNW/PET composite, (**c**)—sheet resistance/thickness comparison [35,36,37,38,39,40,41,42].

A schematic representation of the four-probe sheet resistance measurement method is shown in Figure 4a.

Figure 4b shows the dependence of sheet resistance of AgNW films before and after laser treatment with energy of 0.30 J per pulse (0.19 J/cm^2^). As we said earlier, laser treatment leads to the fusion of AgNW at their contact points and, as a consequence, to an increase in the contact spot, which leads to a drop in resistance after laser treatment. This effect is particularly noticeable in films with minimal AgNW content per unit area. From surface concentrations above 50 μL/cm^2^ we almost observe a plateau in resistance.

We see that the resistance drops with increasing thickness for both treated and untreated films, which follows from Ohm’s law. It is also possible to achieve low sheet resistance using this approach. However, this strategy is not suitable for flexible electronics applications because thick conductive coatings will affect the flexibility of the products. From this point of view, it is worthwhile to compare conductive coatings and films in terms of surface resistivity and coating thickness.

We also calculated the resistivity of the composite, which in the case of an ideal homogeneous film should remain constant and be equal to the resistivity of crystalline silver. In Figure 4b, we see that in our case, the resistivity close to the resistivity of crystalline silver is demonstrated by samples with a thickness of 4.1 ± 0.2 μm (75 μL/cm^2^) and 3.1 ± 0.2 μm (62.5 μL/cm^2^). This is due to the inhomogeneity of our AgNW conductive layer. As the thickness of the conductive layer decreases, the resistivity increases. This is due to the increase in AgNW conductive layer inhomogeneity at small thicknesses.

In Figure 4c we can see the comparison of conductive coatings and films with our work [35,36,37,38,39,40,41,42]. Note that for the whole range of thicknesses, our results are superior to most of the results mentioned in the literature and better than all the results obtained with nanoscale silver. Only the results of Park et al. with imprinted Au NP are superior to our AgNW/PET composites. Laser allows for much better results than inkjet and lithography, and the technology is more cost-effective than lithography, more environmentally friendly, and easily scalable [43].

### 3.4. Electromagnetic Shielding Performance of AgNW/PET Composite

The purpose of EMI shielding is to encapsulate the area to be shielded with a shielding sheath of some conductive or magnetic material to provide EM insulation. The first purpose is to limit the leakage of EM energy outside the area and its effect on external equipment. The second purpose is to prevent EM energy from outside the area from entering the area and affecting internal equipment [44]. Quantitatively, the shielding efficiency is evaluated by the shielding efficiency SE. This is defined in decibels (dB) according to the formula:

SE = 20 lg E_1_/E_2_ (1)

where E_1_ is is the electromagnetic wave (EMW) amplitude at an arbitrary point of the shielding space without the screen and E_2_ is the EMW amplitude at the same point with the screen. For a material, the SE can be interpreted as the inverse of the transmittance S_21_ (dB):

SE = −10lg(S_21_) (2)

**Figure 5:**
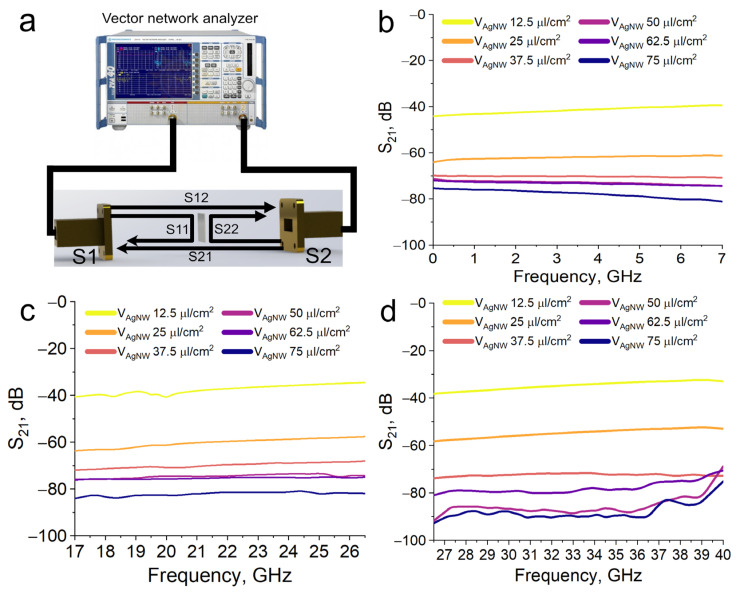
Electromagnetic compatibility. (**a**)—Waveguide method illustration, (**b**)—S_21_ parameter at 0.01–7 GHz range, (**c**)—S_21_ parameter at 17–26.5 GHz range, (**d**)—S_21_ parameter at 26.5–40 GHz range.

Figure 5 shows the dependence of the transmittance coefficient (S_21_) in all investigated frequency ranges (0.01–40 GHz). The SE increases with increasing surface density of AgNW deposited from the base dispersion. The shielding efficiency increases from 40 dB to 90 dB when the surface density increases from 12.5 μL/cm^2^ to 75 μL/cm^2^ respectively. It can be seen that samples with concentrations of 12.5 and 25 mL/cm^2^ tend to decrease in shielding efficiency with increasing frequency. This is due to the fact that laser-treated thin films are structures containing dielectric and conducting regions [44]. The influence of inhomogeneity increases with increasing frequency, which creates a specific slope of the curves for these samples. However, in the context of shielding values, this weak slope does not have a significant effect.

At surface densities of 37.5–75 μL/cm^2^, we see a trend of increasing shielding efficiency with increasing frequency. Thus, in films with a surface density of 37.5 μL/cm^2^, the shielding efficiency shows a weak increase from 70 to 74 dB and 75 µL/cm^2^ film shows an increase in shielding efficiency from 77 dB to 90 dB. The increase in shielding efficiency with increasing frequency for high continuity films is attributed to the skin effect.

The interaction between incident electromagnetic waves and the surface/interface of an electromagnetic shield can be divided into reflection (R), absorption (A), multiple reflection, and transmission (T). The coefficients are calculated using parameters S_11_ and S_21_. The formulas are described below:

R = P_r_/P_i_= 10^0.1S11^ · 100%, (3)

T = P_t_/P_i_= 10^0.1S21^ · 100%, (4)

A = 100% − T − R,(5)

where P_i_, P_r_, P_t_ are the incident, reflected, and transmitted wave powers. The scattering matrix parameters S_11_ and S_21_ (the scattering matrix parameter, which is the transmission coefficient) should be taken in dB.

**Figure 6:**
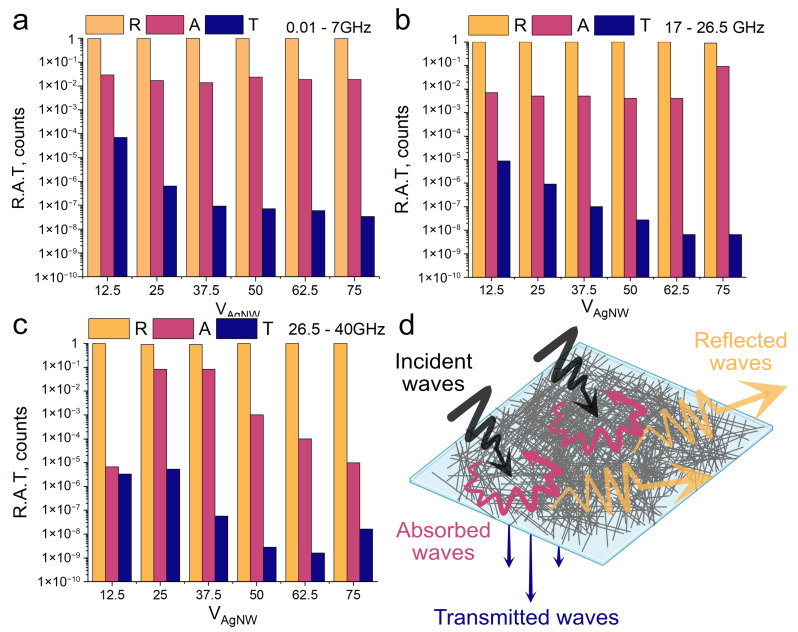
Reflection, absorption, transmission (RAT) diagrams. (**a**)—RAT at 0.01–7 GHz, (**b**)—RAT at 17–26.5 GHz, (**c**)—RAT at 26.5–40 GHz, (**d**)—AgNW/PET composite EMI shielding illustration.

It is possible to determine the energy balance for these three components that make up the interaction of the electromagnetic wave with the screen. Using the Formulas (3)–(5), we calculated these components and made the reflection, absorption, transmission (RAT) diagram (Figure 6). The results of the calculations are shown in Figure 6a–c. Since there is a weak tendency for the shielding efficiency to vary up or down, we averaged the RAT values in the following ranges: 0.01–7 GHz, 17–26.5 GHz, and 26.5–40 GHz and listed the corresponding results as columns. We can conclude that the main mechanism of shielding is reflection. The scheme of the interaction of radio waves with AgNW/PET composite is shown in Figure 6d. The increase of transmittance components with increasing frequency is related to the morphology of AgNW/PET composite. It is a porous structure which can be described as a network. Examples of mesh structures with similar transmittance component growth have already been mentioned in the literature [45].

Modern science places extremely stringent demands on materials. In particular, shielding materials must not only have a high shielding capacity, but also be flexible and, more importantly, thin. In view of such requirements, we have compared those mentioned in the literature with ours, correlating shielding effectiveness with thickness.

**Figure 7:**
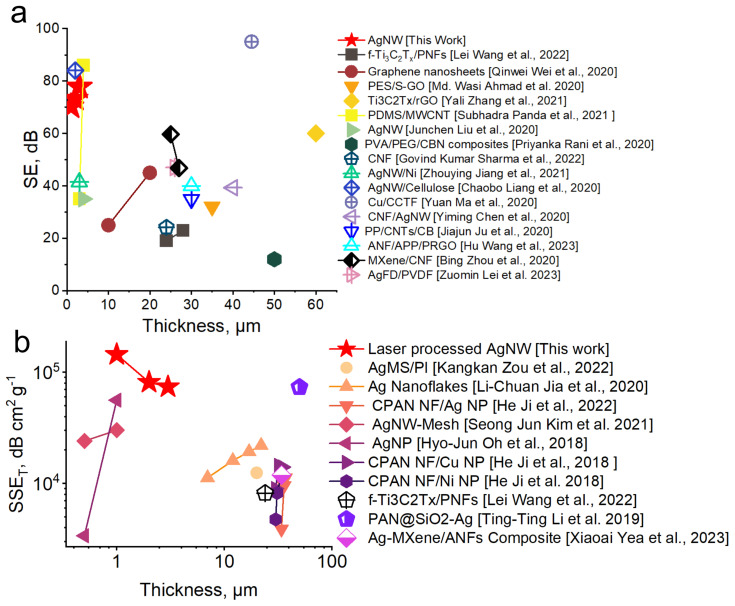
AgNW/PET composite comparisons with alternative materials. (**a**)—SE comparison [45,46,47,48,49,50,51,52,53,54,55,56,57,58,59,60,61], (**b**)—SSE_T_ comparison [61,62,63,64,65,66,67,68].

In Figure 7 you can see the graph. It can be seen that our AgNW/PET composite has a shielding efficiency superior to the coatings mentioned in the analysis. The graphene nanosheet coatings obtained by Panda et al. and Yuan et al. surpass our results, but only at thicknesses many times greater than ours [45,46,47,48,49,50,51,52,53,54,55,56,57,58,59,60].

In general, similar results can be achieved using ordinary thick materials. However, their high density and high weight are not suitable for aerospace, telecommunication applications, and materials for wearable and flexible electronics.

To evaluate the shielding efficiency, taking into account the thickness of the screen, the parameter of specific shielding efficiency (SSE) is used, which is calculated by the following formula [44]:

SSE_t_ = SE_T_/ρ × t (6)

where ρ—density in g/cm^3^ and t—thickness in mm.

In Figure 7b, you can see a graph comparing SSE for different manuscripts. In the comparison of SSE_t_ values, we used only metallic nano-objects of different shapes (nanowires, nanoplates, nanoparticles, etc.). As we can see, our coating shows one of the best results for thicknesses below 5 microns.

### 3.5. Mechanical Properties AgNW/PET Composite

**Figure 8:**
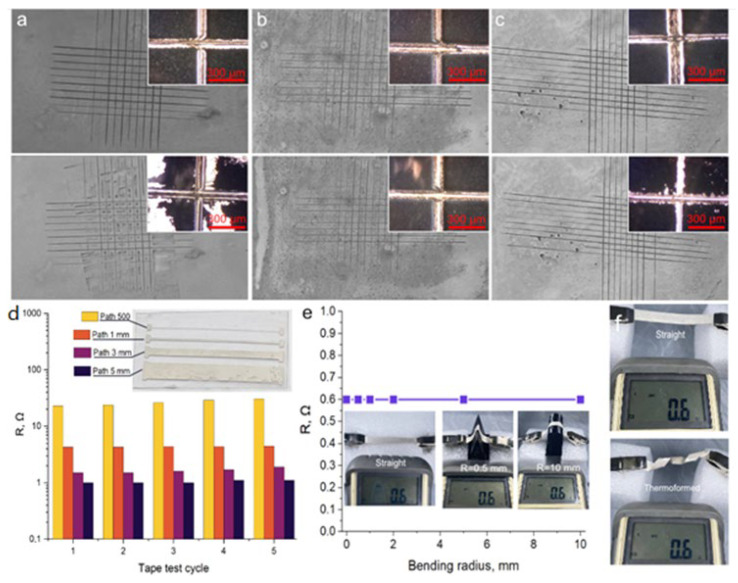
Mechanical properties. (**a**)—Optical image of untreated film before and after the ASTM D3359 test; (**b**)—optical image of processed film with C_AgNW_ = 12.5 μL/cm^2^ before and after the ASTM D3359 test; (**c**)—optical image of processed film with C_AgNW_ = 75 μL/cm^2^ before and after the ASTMD D3359 test; (**d**)—change in resistance after a tape test; (**e**)—changing of resistance at different bending radius; (**f**)—thermoforming demonstration.

Flexible electronics involves the use of materials under a load that bends or curls the product. We have conducted various mechanical stability tests to evaluate the suitability of our composites for flexible electronics applications. The results of these tests are shown in Figure 8.

Figure 8a–c shows optical photographs of AgNW films before laser treatment and after laser treatment. The images of maximum and minimum concentrations (12.5 µL/cm^2^ and 75 µL/cm^2^) for laser treated films are also shown. Adhesion was tested according to ASTM D3359. The adhesion of the films without laser treatment is at 0–1B, which means a peel percentage of 35–65%. This level of adhesion is not suitable for use in flexible electronics applications. The treated films show adhesion level 5B for concentrations of 12.5–50 µL/cm^2^ and 4B for 62.5 and 75 µL/cm^2^. The decrease in adhesion level is due to the fact that the large thickness (about 4 μm) prevents the sintering of AgNW located in different planes. The high adhesion of AgNW to the substrate indirectly confirms the formation of a composite at the PET/AgNW interface.

We tested AgNW/PET composites for resistance to tape tear-off. We fabricated a conductive pattern, in the form of paths with different widths (500 µm, 1 mm, 3 mm, and 5 mm), at the ends of which were contact pads for measuring equipment (Figure 8d). The results of resistance changing after each detachment are shown in the histogram in Figure 8d. Each of the conductors retained electrical conductivity after five cycles of the taping test. The resistance of the 500 µm track changed the most. Removal of the same amount of AgNW from paths of different widths left different amounts of surviving contacts, which is associated with the increase in resistance.

High adhesion of the conductive layer to the substrate is one of the main requirements for flexible electronics. However, it is not the only one. The material must be able to retain its properties after bends of various radii. To test the durability of the AgNW/PET composite, we performed bending tests with radii of 0.5 mm, 1 mm, 2 mm, 5 mm, and 10 mm. Figure 8e shows the graph of resistance variation as a function of bending radius. The resistance remained at the same level regardless of the bending radius. The sintered contacts between single AgNW after laser treatment retained high tensile strength.

Flexible electronics involves the constant bending and unbending of devices, which requires materials to be resistant to fatigue damage. To test the durability of AgNW/PET composites, we tested them for 1000 cycles of bending-extension with a radius of 0.5 mm. We measured the resistance of the AgNW/PET composite after every 100 bends (Figure S3).

AgNW/PET composite retains the thermoplastic substrate’s thermoforming ability, without loss of electrical properties. This expands the scope of its potential applications and makes it possible to use it in various technological processes [69]. Demonstration of the thermoforming process together with detailed characterization of mechanical properties provides a basis for the integration of such composites into industry.

## 4. Conclusions

In this work, we demonstrated the laser sintering of AgNW with their subsequent integration into the substrate and formation of AgNW/PET composites. In the UV-Vis spectroscopy, we found absorption peaks at 345 and 410 nm, respectively. These two peaks are related to the plasmon surface resonance phenomenon. Considering this, we made AgNW films on a polymer substrate and treated them with a laser with a wavelength of 432 nm. After treatment, we characterized our films by SEM, EDX, and XRD and measured the sheet resistance. From the results, we found that the AgNW were heated by the laser exposure, which led to their sintering or melting, and the melting of the surface of the polymer substrate, followed by the integration of wires and the formation of a composite. The obtained AgNW/PET composite has a low sheet resistance of about 30 mΩ/sq. We also conducted shielding efficiency measurements. Our composite is capable of shielding in wide frequency ranges at the level of 89 dB with thickness about 3.1 ± 0.2 μm. In order to fully evaluate the applicability of the obtained material in flexible electronics applications, we conducted comprehensive mechanical tests, including an ASTM D3359 test, tape test, bending resistance test, cyclic bending test, and thermoforming. As a result, the AgNW/PET composite demonstrated high adhesive strength and bending resistance, including cyclic loads, while maintaining the ability to thermoform. The results obtained are an important addition to the field of manufacturing thermoplastic polymer composites for flexible electronics applications.

## Data Availability

Data is contained within the article or Supplementary Materials.

## Supplementary Materials

The following supporting information can be downloaded at: https://www.mdpi.com/article/10.3390/polym16223174/s1, Figure S1: Photo before and after washing off excess AgNW after laser treatment; Figure S2: Search for modes for laser processing. The figure shows that we can only work in a narrow range of laser speeds and power. 100% speed corresponds to 100 mm per minute; Figure S3: Cyclic load for 1000 cycles. Measurements were taken every 100 bending-extension cycles; Video S1: Sample washing process.
