# Supplementary material for: Laser-Induced Silver Nanowires/Polymer Composites for Flexible Electronics and Electromagnetic Compatibility Application
Source: Polymers (Basel). 2024 Nov 14;16(22):3174. doi: 10.3390/polym16223174 (PMC11598760; doi:10.3390/polym16223174)
Supplement: Supplementary file 1 [file polymers-16-03174-s001.zip › Supporting information.pdf]

Laser induced silver nanowires/polymer composites for flexible electronics and electromagnetic compatibility application.

Il'ya Bril[1*], Anton Voronin[1,2,3], Yuri Fadeev[1], Alexander Pavlikov[3], Ilya Govorun[4,5], Ivan Podshivalov[4], Bogdan Parshin[2], Mstislav Makeev[2], Pavel Mikhalev[2], Kseniya Afanasova[1,6], Mikhail Simunin[1,2,3], and Stanislav Khartov[1]

Supporting information

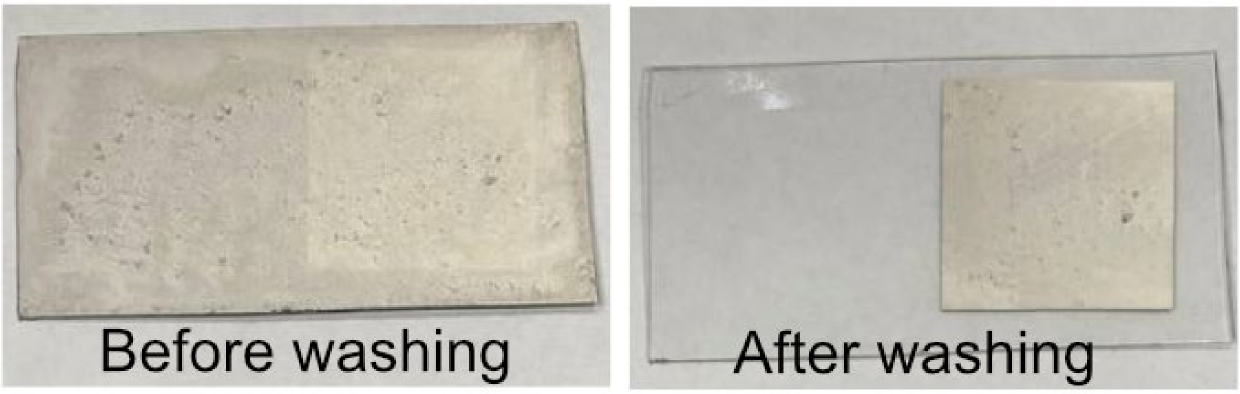

Figure S1. Photo before and after washing off excess AgNW after laser treatment.

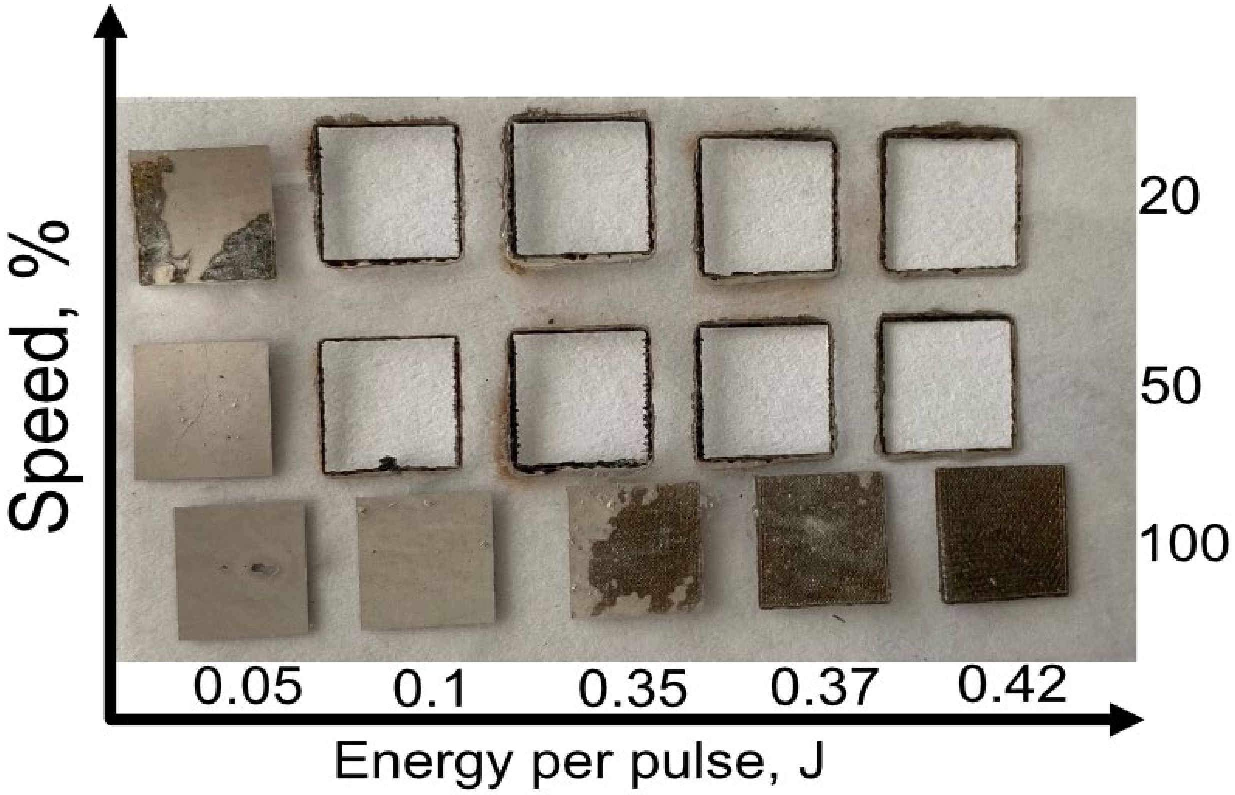

Figure S2. Search for modes for laser processing. The figure shows that we can only work in a narrow range of laser speeds and power. 100% speed corresponds to 100 mm per minute.

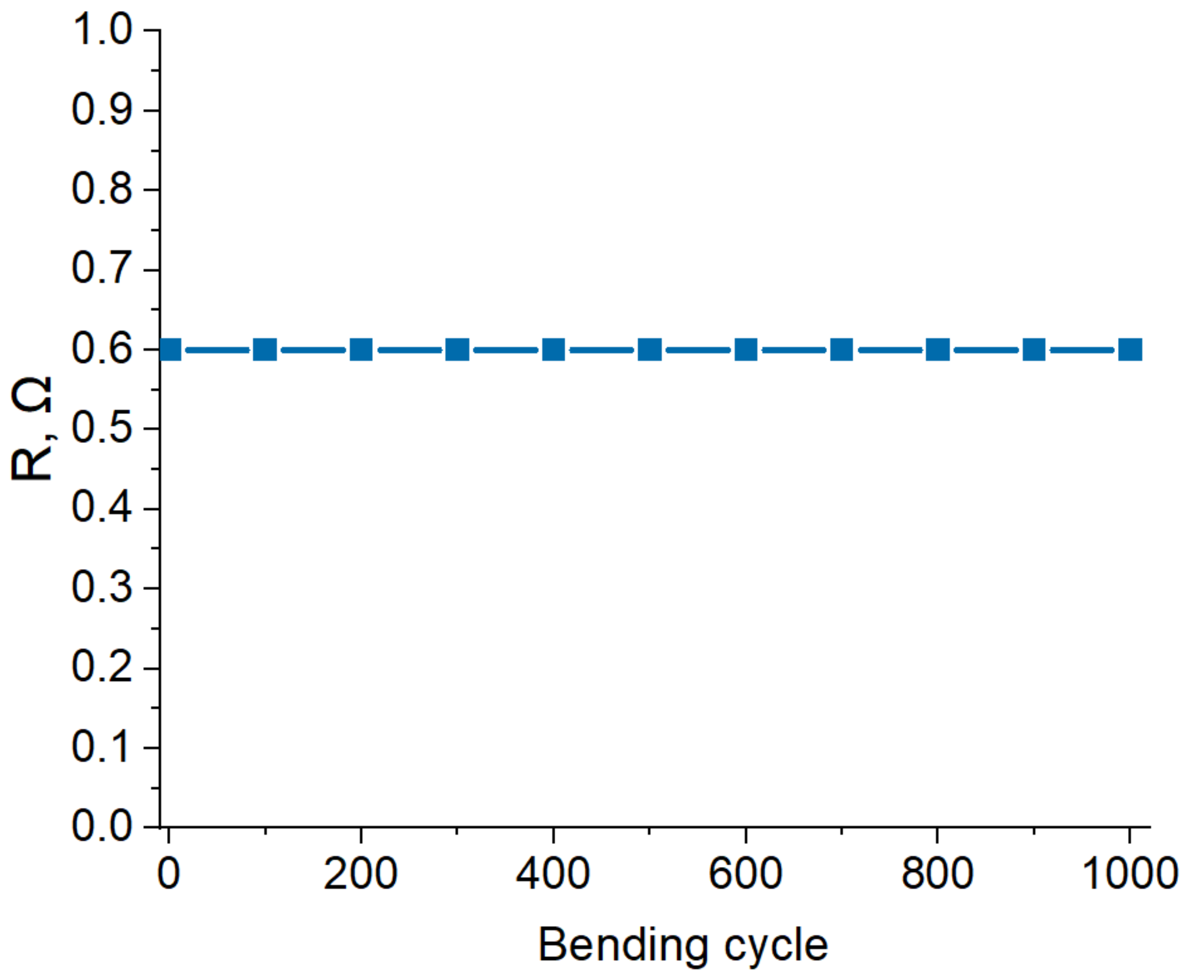

Figure S3. Cyclic load for 1000 cycles. Measurements were taken every 100 bending-extension cycles
